# Generating HPV specific T helper cells for the treatment of HPV induced malignancies using TCR gene transfer

**DOI:** 10.1186/1479-5876-9-147

**Published:** 2011-09-05

**Authors:** Kirsten BJ Scholten, Annelies W Turksma, Janneke J Ruizendaal, Muriel van den Hende, Sjoerd H van der Burg, Mirjam HM Heemskerk, Chris JLM Meijer, Erik Hooijberg

**Affiliations:** 1 Department of Pathology, VU University Medical Center, de Boelelaan 1117, 1081 HV Amsterdam, The Netherlands; 2 Department of Gynecology, Leiden University Medical Centre, Albinusdreef 2, 2300 RC Leiden, The Netherlands; 3 Department of Clinical Oncology, Leiden University Medical Centre, Albinusdreef 2, 2300 RC Leiden, The Netherlands; 4 Department of Hematology, Leiden University Medical Centre, Albinusdreef 2, 2300 RC Leiden, The Netherlands

## Abstract

**Background:**

Infection with high risk Human Papilloma Virus (HPV) is associated with cancer of the cervix, vagina, penis, vulva, anus and some cases of head and neck carcinomas. The HPV derived oncoproteins E6 and E7 are constitutively expressed in tumor cells and therefore potential targets for T cell mediated adoptive immunotherapy. Effective immunotherapy is dependent on the presence of both CD4+ and CD8+ T cells. However, low precursor frequencies of HPV16 specific T cells in patients and healthy donors hampers routine isolation of these cells for adoptive transfer purposes. An alternative to generate HPV specific CD4+ and CD8+ T cells is TCR gene transfer.

**Methods:**

HPV specific CD4+ T cells were generated using either a MHC class I or MHC class II restricted TCR (from clones A9 and 24.101 respectively) directed against HPV16 antigens. Functional analysis was performed by interferon-γ secretion, proliferation and cytokine production assays.

**Results:**

Introduction of HPV16 specific TCRs into blood derived CD4+ recipient T cells resulted in recognition of the relevant HPV16 epitope as determined by IFN-γ secretion. Importantly, we also show recognition of the endogenously processed and HLA-DP1 presented HPV16E6 epitope by 24.101 TCR transgenic CD4+ T cells and recognition of the HLA-A2 presented HPV16E7 epitope by A9 TCR transgenic CD4+ T cells.

**Conclusion:**

Our data indicate that TCR transfer is feasible as an alternative strategy to generate human HPV16 specific CD4+ T helper cells for the treatment of patients suffering from cervical cancer and other HPV16 induced malignancies.

## Background

Human Papilloma Viruses (HPV) play an important role in the development of cervical cancer (CxCa), which is the second most common cause of cancer related deaths among women world-wide. Each year approximately 500,000 women, commonly between the ages of 30 and 50, are diagnosed with this type of cancer[1]. Other less common cancers associated with HPV infections are cancers of the vulva[2], vagina[3], anus[4], penis[5] and some cases of head and neck cancers[6].

Although the incidence of cervical cancer has been decreased by population based screening in the Western world, new cases of cervical cancer still occur. The success of treatment by surgery, radiotherapy, chemotherapy or a combination there-of is often high in lower stages of the disease but decreases in higher stages. A reduction in the number of cervical cancer patients is to be expected since two prophylactic vaccines, Gardasil and Cervarix, have been implemented in a number of countries around the world. However, till this point it is unclear whether these vaccines are protective against HPV induced malignancies other than cervical cancer. Therefore, other methods to treat patients suffering from cervical cancer and HPV induced malignancies should be explored.

Adoptive transfer of HPV specific T cells could be an attractive strategy to treat patients suffering from HPV induced malignancies. The HPV16-derived oncoproteins E6 and E7, responsible for both onset and maintenance of malignant transformation, are constitutively expressed in HPV induced cancers and represent non-self tumor-associated antigens. As such the HPV antigens E6 and E7 are high on the priority-ranked list of cancer vaccine target antigens[7] In previously described clinical trials applying adoptive transfer of melanoma specific CD8+ T cells, no objective clinical responses were found in melanoma patients while melanoma specific CD8+ T cells were highly reactive against tumor cells in vitro[8]. More recent clinical trials using both CD4+ and CD8+ T cells were more successful since 18 out of 35 patients showed a clinical response, including three complete responders[9,10]. Therefore, adoptive transfer of HPV specific CD4+ and CD8+ T cells might be attractive to treat patients suffering from HPV induced malignancies. However, CTL responses against HPV antigens in women with natural HPV infections are difficult to detect indicating that the precursor frequencies of HPV specific CTLs are very low, making it difficult to isolate these HPV specific T cells[11,12].

In addition, HPV16 specific CD4+ T helper responses were either absent or severely impaired in patients with HPV16 positive genital lesions and patients suffering from cervical cancer[13-15]. Increased numbers and activity of HPV specific CD4+ and CD8+ T cells can be found in cervical cancer patients after vaccination with synthetic long peptides (SLP)[16]. In the majority of patients suffering from premalignant HPV positive vulvar intraepithelial neoplasia regression was observed after SLP vaccination[17]. In contrast, hardly any clinical responses were observed in late stage cervical cancer patients after SLP vaccination[18].

A promising "off the shelf" method to generated high numbers of tumor specific T cells consists of the introduction of antigen specific TCR genes into recipient T cells. Recently, the generation of HPV specific CD8+ T cells was described using both E6 and E7 specific TCRs[19,20]. For the generation of tumor specific CD4+ T cells two different strategies can be applied. First, TCRs derived from CD4+ T cell clones can be introduced into recipient CD4+ T cells as has been described previously for non HPV malignancies[21]. Second, tumor specific CD4+ T cells can be generated by introducing MHC class I restricted TCRs[22]. Previous reports have shown that the introduction of a CD8 independent TCR into CD4+ T cells resulted in production of cytokines after co-culture with peptide pulsed cells and cytotoxicity against tumor cells[22].

In this paper we show that T helper function, as measured by specific cytokine production, against HPV16 antigens can be obtained after transfer of MHC class I or MHC class II restricted HPV specific TCRs into CD4+ T cells.

## Materials and methods

### Cell lines and T cell culture

For the generation of a CD4+ T cell population, healthy donor derived PBMC were isolated from buffycoats by density gradient centrifugation using Lymphoprep (Nycomed, Oslo, Norway). Subsequently, isolation of resting CD4+ helper T cells from total PBMC was performed by positive selection on a magnetic sorting device (MACS; Miltenyi Biotec, Bergisch Galdbach, Germany). For this purpose, total PBMC were stained with anti-CD4 Ab and microbead-conjugated anti-mouse IgG Abs (Miltenyi Biotec), followed by MACS sorting according to the manufacturer's protocols. T cell blasts from a HLA-DP1 matched, non-autologous, donor were obtained by stimulating PBMCs with 800 ng/ml PHA and 100 IU/ml IL-2 for 1 week. T cell blasts and CD4+ T cells were cultured in Yssel's medium[23] supplemented with 1% human serum (HS; ICN Biomedicals, Aurora, OH, USA) and antibiotics (100 IE/ml penicillin and 100 μg/ml streptomycin, Life technologies). T cell blasts and CD4+ T cells were stimulated weekly with an irradiated feeder mixture as has been described previously[19].

The HLA-DP1+ EBV transformed B cell line EBV24 and Jurkat/MA cells were cultured in IMDM supplemented with 8% (v/v) fetal calf serum (FCS; Perbio, Helsingborg, Sweden) and antibiotics. The HLA-A2+, non-autologous, melanoma cell line melAKR was transduced with minigene constructs encoding the HPV16E7_11-20wt _(YMLDLQPETT) or the HPV16E7_11-20V _(YMLDLQPETV) epitope. The latter epitope containing the V-variant was shown previously to bind more efficiently to HLA-A2[24,25]. The resulting model cell lines containing these constructs have been named melAKR-E7wt and melAKR-E7V, respectively. EBV24 is autologous to T cell clone 24.101, whereas Jurkat/MA and melAKR are non-autologous to T cell clone 24.101 and to T cell clone A9. The expression level of HLA-A2 differed substantially between cell lines. FACS analyses using two different HLA-A2 specific antibodies (BB7.2 and B17) showed a mean fluorescence intensity of 900-1000 for melAKR and of 1300-1400 for JY, which is homozygous for HLA-A2. Cervical carcinoma cell lines like Caski and CxCa866 typically showed an MFI of 200-300[25]. All cells were tested mycoplasm free and were maintained at 37°C in humidified air containing 5% CO_2_

### DNA constructs

In order to isolate the TCR open reading frames from CD4+ T cell clone 24.101, total RNA was isolated from 1.5 × 10^6 ^cells and PCR was performed as has been described earlier[19]. PCR products were ligated into the pCR2.1 vector (Invitrogen). Sequence analysis was performed to determine TCRα and TCRβ usage of this particular T cell clone (BaseClear, Leiden, the Netherlands). As has been shown previously, TCR expression can be greatly enhanced after codon-modification of the TCR ORFs[26,27] or the introduction of an extra cysteine in the constant domain of both the TCRα and TCRβ chains[28]. The first improves greatly on translation into protein whereas the latter facilitates binding of the exogenous TCRα and TCRβ chains. Therefore, TCR ORFs of clone 24.101 were codon-optimized and an extra cysteine was incorporated in the constant domains (GeneArt, Regensburg, Germany). Wild-type and codon-modified plus cysteinized (from here on called cmCys) TCRα and TCRβ ORFs were cloned into the multiple cloning site (mcs) of the Moloney murine leukemia based retroviral vector LZRS, followed by the marker GFP or the truncated version of the nerve growth factor receptor (NGFR), respectively[29], resulting in LZRS-TCRα(24.101)-IRES-GFP and LZRS-TCRβ(24.101)-IRES-NGFR. For experiments with the codon modified A9 TCR the retroviral construct carrying cmTCRα-2A-cmTCRβ was used, which has been described previously in detail[26]. We also used a retroviral LZRS construct encoding CD8α-2A-CD8β in A9-TCR transgenic CD4+ T cells to examine differences in TCR expression and functional activity.

The sorting signal of lysosome associated membrane protein-1 (LAMP-1) reroutes antigens to the MHC class II processing pathway, resulting in enhanced presentation to CD4+ T cells in vitro[30]. To accomplish enhanced presentation of HPV16E6 we linked LAMP-1 to HPV16E6 resulting in sig-[HPV16E6LAMP]. This DNA fragment was introduced into the multiple cloning site of the retroviral vector, LZRS-mcs-IRES-GFP.

### Retroviral transduction and analysis of gene expression

All retroviral constructs were transfected into the packaging cell line Phoenix-a using lipofectamine (Invitrogen), two days after transfection 2 μg/ml puromycin (Sigma-Aldrich, St. Louis, MO) was added. Ten to 14 days after transfection puromycin free retroviral supernatant was harvested.

T cell blasts were retrovirally transduced with either GFP or sig-[HPV16E6LAMP]-IRES-GFP. Subsequently, T cell blasts were sorted for the expression of GFP using flow cytometry. Retroviruses encoding for the TCR ORFs were used to transduce Jurkat/MA and CD4+ T cells as has been described previously[19,31]. Retrovirus encoding for CD8 was used to transduce TCR transgenic CD4+ T cells.

Expression of the TCR was determined after 48h and later time points by flow cytometric analysis. Antibodies used were FITC labeled antibodies directed against CD8, PE labeled antibodies directed against human TCRVβ3, TCRVβ17 and TCRVβ1, and allophycocyanin labeled anti-human nerve growth factor receptor (NGFR; Chromoprobe, Aptos, CA) antibody. APC-labeled HLA-A2.1 tetramers presenting the HPV16E7_11-20 _epitope were used[25]. Tetramer and Ab staining of cells was performed in PBS supplemented with 0.1% BSA and 0.01% azide (PBA) for 15 min at 37°C and 20 min on ice respectively, followed by washing with PBA. Stained cells were analyzed on a FACSCalibur (BD Biosciences, San Jose, CA, USA) using CellQuest software (BD Biosciences). 24.101 TCR transgenic CD4+ T cells expressing GFP and NGFR were isolated by NGFR/GFP directed flow sorting in complete medium. In addition, A9 TCR transgenic T cells positive for tetramers were sorted by tetramer directed flow sorting while those also carrying CD8 were sorted based on tetramer binding and CD8 expression.

### Functional read-out assays

HPV16E7_11-20_, HPV16E6_73-104 _and irrelevant (HPV16E6_37-68 _and MP1_58-66_) peptides were synthesized by the Leiden University Medical Center (LUMC, the Netherlands). Peptides were >90% pure as analyzed by reverse-phase HPLC, dissolved in DMSO and stored at -20°C until further use.

Functionality of TCR transgenic Jurkat/MA cells was measured using a luciferase assay[32]. To measure the activation of 24.101 TCR transduced Jurkat/MA cells by EBV24 cells loaded with 10 μM of irrelevant HPV16E6_37-68 _peptide or relevant HPV16E6_73-104 _peptide, 10^5 ^Jurkat/MA cells were incubated overnight with 5 × 10^4 ^target cells in a 96-well plate. After incubation with various stimuli, cells were analyzed for luciferase activity. Luminescence was subsequently measured in a Lumat LB 9507 luminometer (EG and G Berthold, Bad Wildbad, Germany). Luciferase activity in stimulated Jurkat/MA cells was expressed as relative luminescence units (RLU) related to the luciferase activity of non-stimulated Jurkat/MA cells, which was set at a value of one.

Production of interferon-γ by stimulated CD4+ T cells was determined using intracellular interferon-γ staining of permeabilized T cells with PE labeled IFN-γ specific antibody according to the manufacturer's instructions (Cytofix/Cytoperm kit with golgistop, BD Bioscience). MHC class-II DP1 matched, non-autologous, monocytes or monocyte derived dendritic cells were pulsed overnight at 37°C with 10 μg/ml peptide or 20 μg/ml protein in serum free medium. After this incubation cells were washed extensively to remove excess peptide or protein prior to subsequent experiments. Stimulations were next performed for 4 hrs at 37°C in a round bottom 96-well plate (Nunc) containing 1 × 10^5 ^responder CD4+ T cells and 5 × 10^4 ^target cells per well, followed by either NGFR staining or CD8/tetramer staining as described above, and intracellular IFN-γ staining. Samples were subsequently analyzed by flow cytometry in order to calculate the percentage of responding CD4+ T cells.

Proliferation assays were performed by stimulating 10^4 ^TCR transgenic CD4+T cells with 10^3^-10^4 ^target cells for 4 days in 96-well plates in Yssel's medium. At day 4, 2.5 μCi/ml [^3^H]-thymidine (Amersham, Aylesbury, UK) was added per well for a period of 16 hours. Plates were harvested onto glass fiberglass filters. Incorporation of [^3^H] thymidine was quantified using a Topcount NXT Microbetacounter (Packard, Meriden, CT).

In order to measure cytokine production upon specific stimulation a Cytokine Bead Array (CBA) assay was performed using the manufacturer's protocol (BD Biosciences, San Jose, CA, USA). Typically 1 × 10^5 ^CD4+ T cells were co-cultured with 5 × 10^4 ^target cells in a round bottom 96-well plate (Nunc). After 24 hours of incubation, cell-free supernatants were harvested and stored at -20°C until CBA quantization.

## Results

### Isolation and preservation of a MHC class II restricted HPV16E6 specific TCR

As a source of MHC class II restricted HPV specific TCRs we used T cell clone 24.101. This HPV16E6_73-104 _specific CD4+ T cell clone 24.101 was generated from a healthy donor after short term in vitro stimulation with peptide, followed by limiting dilution cloning[33]. This helper T cell clone was shown to recognize the HLA-DP1 restricted HPV16E6_73-104 _T cell epitope as a synthetic peptide but also as an endogenously processed and presented epitope[34]. Flow cytometric analysis showed that this T cell clone was TCRVβ17 positive which was confirmed by sequence analysis. Sequence analysis also revealed TCRVα1 usage (Table 1).

**Table 1:** TCR CDR3 region of the HPV16E6 specific CD4+ T cell clone 24.101

TCR family	V region	NDN region	J segment	C region
**TCR**β**17**	TGT GCC AGT AGT	TAC CAA GGG AGC TCT	GGA AAC ACC ATA TAT T TT GGA GAG GGA AGT	TGG CTC ACT GTT GTA GAG GAC
	C A S S	Y Q G S S	G N T I Y F G E G S	W L T V V E D

**TCR**α**1**	GAG TAC TTC TGT	GCT GTG GGC CCA	AAT ACT GGA GGC TTC AAA ACT ATC TTT GGA GCA GGA AC A AGA CTA	TTT GTT AAA GCA AAT ATC CAG
	E Y F C	A V G P	N T G G F K T I F G A G T R L	F V K A N I Q

T cell receptor variable domains were designated according to Arden et al in Immunogenetics (1995) 42:455-500.

**Figure 1:**
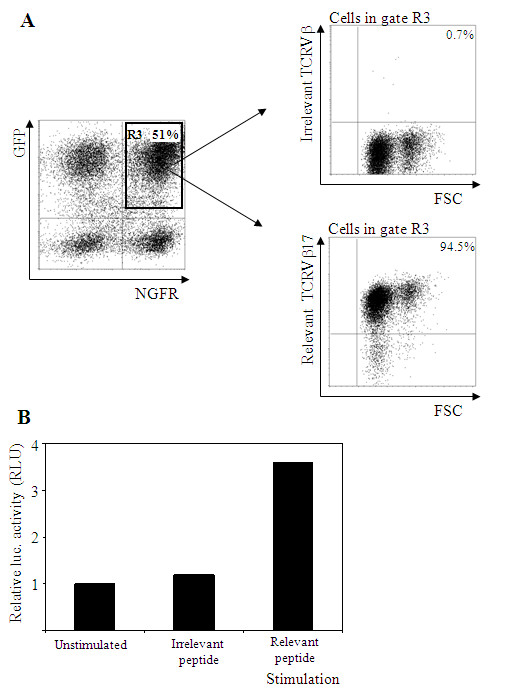
**Phenotypic analysis and functional activity of wildtype 24.101 TCR specific for HPV16E6_(73-104) _in TCR transgenic Jurkat/MA cells**. (A) Jurkat/MA cells transduced with vectors LZRS-wtTCRα-I-GFP and LZRS-wtTCRβ-I-NGFR were analyzed for GFP and NGFR marker gene expression by flow cytometry. Transduced cells co-expressing GFP and NGFR (gate R3) were negative for irrelevant TCRVβ (upper right plot) but positive for relevant TCRVβ17 (lower right plot) staining, as indicated by the percentage TCRβ positive cells (upper right quadrant). (B) Functional activity of 24.101 TCR transgenic Jurkat/MA cells as determined in a luciferase assay in response to stimulation with the HLA-DP1 positive cell line EBV24, exogenously loaded with relevant HPV16E6_(73-104) _or irrelevant HPV16E6_(37-68) _peptide. Luciferase activity in Jurkat/MA cells is shown in Relative Luminescence Units (RLU), defined as the ratio of luciferase activity in stimulated versus unstimulated cells Data are shown from one representative experiment out of two performed.

Isolated TCRα and TCRβ ORFs from CD4+ T cell clone 24.101 were cloned into the marker gene containing retroviral vector LZRS as TCRα-IRES-GFP and TCRβ-IRES-NGFR. To investigate proper formation of stable TCRαβ complexes on the cell surface, we introduced the TCRα and TCRβ chain into the reporter cell line Jurkat/MA. Surface expression of the transgenic TCR at the cell surface was determined using TCRVβ antibodies, since tetramers or multimers containing the epitope were unavailable. Co-expression of the marker genes GFP and NGFR was found in approximately 51% of the transduced Jurkat/MA cells (Figure 1A, left panel). In the GFP+/NGFR+ gate ~95% of the cells were positive for TCRVβ17 staining (Figure 1A, right hand lower panel). In the same gate only background staining was observed using irrelevant TCRVβ antibodies (Figure 1A, right hand upper panel). Jurkat/MA cells do not express an endogenous TCRβ chain but do express a TCRα chain. Therefore, we analyzed whether the TCRβ chain is capable of cross-pairing with this particular TCRα chain. Some background TCRVβ17 staining was detected in the GFP-/NGFR- (~2%) quadrant. In the GFP-/NGFR+ quadrant ~8% of the cells were positive for TCRVβ17 staining (data not shown). This clearly indicates that the introduced TCRVβ17 does not cross-pair to high levels with the TCRVα8 endogenously present in Jurkat/MA cells. Moreover the TCRVβ17 is very well expressed in GFP/NGFR transgenic Jurkat/MA T cells where there is no competition from other TCRVβ chains for components of the CD3 complex.

Functional activity was measured using a luciferase assay. TCR transgenic Jurkat/MA cells were stimulated overnight with the HLA-DP1+ EBV24 cell line, which was derived from the same donor as the 24.101 T cell clone, exogenously loaded with irrelevant peptide or relevant HPV16E6_73-104 _peptide. As depicted in Figure 1B, specific luciferase activity could be detected after stimulation with EBV24 exogenously loaded with the relevant peptide. No luciferase activity could be detected after stimulation with irrelevant peptide. From these results we concluded that we successfully isolated the correct TCRα and TCRβ pair from T cell clone 24.101 and that this TCR is functionally active as determined in the Jurkat/MA-NFAT-luciferase system.

### MHC class II restricted HPV16E6 specific TCR transgenic CD4+ T cell: TCR expression and functionality

**Figure 2:**
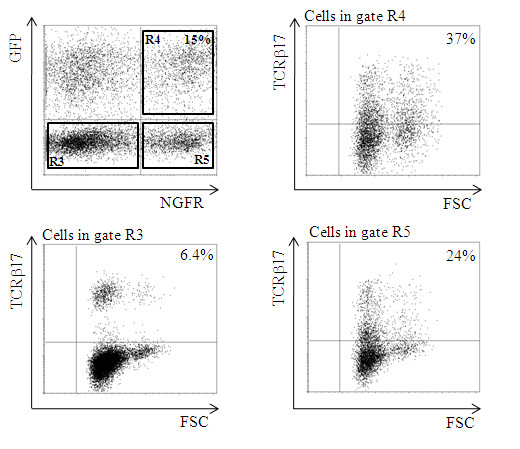
**TCRVβ17 expression analysis of codon-modified/cysteinized HPV16E6_(73-104) _specific 24.101 TCR transgenic CD4+ T cells**. Isolated CD4+ T cells transduced with vectors cmCysLZRS-TCRα1-IRES-GFP and cmCysLZRS-TCRβ17-IRES-NGFR were analyzed for GFP and NGFR marker gene expression by flow cytometry. Cells in the GFP/NGFR double negative quadrant (Gate R3) staining positive for TCRVβ17 represent the TCRβ17+ cells endogenously present in this CD4+ population. In de GFP/NGFR double positive gate R4 37% stain with the TCRVβ17 antibodies. About 6.4% represent the endogenously population, hence about 30% of the cells appear to be TCR transgenic. Part (6.4%) of the T cells in gate R5 stained positive with TCRVβ17 antibodies, again representing the endogenously present population. About 18% (24% minus 6.4% endogenous) of the TCRVβ17 positive cells are transgenic for the TCRVβ17-IRES-NGFR construct. Thus clearly showing that the TCRVβ17 was capable of pairing with endogenously present TCRα chains. Results shown here are representative for three different donors tested.

To investigate the application potential of HPV16E6 specific CD4+ T cells, we tested the expression of the HPV16E6_73-104 _specific TCR in human peripheral blood derived CD4+ T cells of unrelated donors. For this purpose we introduced the cmCys 24.101 TCR ORFs into CD4+ T cells. Transduced CD4+ T cells were analyzed for the expression of GFP and NGFR. As shown in Figure 2 co-expression of the markers was found in 15% of the CD4+ T cells. Since no tetramers or multimers were available to visualize the antigen specific TCR transgenic T cells, cell surface expression of the introduced TCR was analyzed using TCRVβ17 antibodies. In the GFP/NGFR double negative gate 6.4% of the cells were positive for TCRVβ17 staining, representing the percentage of TCRVβ17+ cells naturally present in this donor. Importantly, in the GFP+/NGFR+ gate R4, 37% of the cells were positive for TCRVβ17 staining. Non transgenic T cells with high endogenous expression of TCRVβ17 can be identified in gate R4 and in gate R5. In the GFP-/NGFR+ gate R5, 24% of the cells were positive for TCRVβ17 staining (Figure 2, gate R5). About one quarter of the cells in R5 have high expression of endogenous TCRVβ17, where the other three quarter is transgenic. This clearly indicates that this 24.101 TCRβ is very promiscuous and capable of pairing with endogenously present TCRα chains. It is unclear why the percentage of TCRVβ17 positive T cells in the GFP/NGFR double positive transgenic T cells was only about 31% (37% minus the endogenous TCRVβ17 T cell population of about 6.4%).

**Figure 3:**
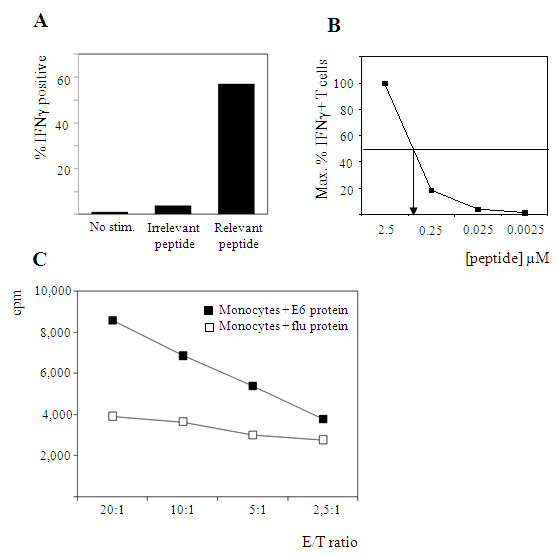
**Functional activity of HPV16E6_(73-104) _specific 24.101 TCR transgenic CD4+ T cells against peptide or protein loaded target cells**. (A) IFNγ production of TCR transgenic CD4+ T cells as determined in an intracellular IFNγ assay. Target cells used were HLA-DP1 matched, non autologous, mature dendritic cells exogenously loaded with relevant HPV16E6_(73-104) _or irrelevant HPV16E6_(37-68) _peptide. Intracellular IFNγ staining is shown for GFP/NGFR double positive T cells. (B) Functional avidity analysis of HPV16E6_(73-104) _specific TCR transgenic CD4+ T cells as determined in an intracellular IFNγ assay. Target cells used were the HLA-DP1 positive cell line EBV24 (autologous to the original 24.101 T cells) loaded with serial 10-fold dilutions of the HPV16E6_(73-104) _peptide. (C) Proliferative capacity of 24.101 TCR transgenic CD4+ T cells as determined in a thymidine incorporation assay. Target cells used were DP1 matched, non-autologous, monocytes loaded overnight with 20 μg/ml relevant E6 or irrelevant flu protein. Results shown in panels A, B and C are representative for three different donors tested.

To measure functional activity, TCR transduced T cells were sorted based on GFP and NGFR expression. The resulting population showed 96% positivity for both GFP and NGFR and again only part (36%) of the T cells were positive for staining with TCRVβ17 antibodies. Functional activity of cmCys TCR transgenic CD4+ T cells against peptide loaded target cells was tested using an intracellular interferon-γ assay. Upon stimulation with HLA-DP1 matched, non-autologous, mature dendritic cells loaded with relevant E6_73-104 _peptide, 57% of the cells were capable of producing interferon-γ (Figure 3A). Low level staining was detected after stimulation with irrelevant peptide (Figure 3A).

A peptide titration experiment was performed using EBV24 cells loaded with decreasing amounts of the HPV16E6_73-104 _peptide. The results, given in Figure 3B, showed that even at the highest concentration used, a plateau was not reached. Hence the 24.101 TCR transgenic CD4+ T cells recognize the HPV16E6_73-104 _peptide with a half maximum interferon-γ production of ~1 μM of added peptide at best, which is indicative of low avidity T cells.

Subsequently, we investigated whether endogenously processed antigen could be recognized by these TCR transgenic CD4+ T cells. Functional activity of TCR transgenic T cells against protein loaded target cells was tested using a proliferation assay. Therefore, monocytes derived from an HLA-DP1 matched, non-autologous donor were pulsed with either relevant HPV16E6 protein or irrelevant flu MP1 protein. As expected, TCR transgenic T cells proliferated specifically upon stimulation with monocytes loaded with E6 protein and not after stimulation with monocytes loaded with flu MP1 protein (Figure 3C).

To investigate cytokine production, thus allowing us to analyze which T helper phenotype (Th1/Th2/Treg) is present in the TCR transgenic CD4+ T cell population, a cytokine bead array assay was performed. For this purpose T cell blasts from an HLA-DP1 positive donor were transduced with either GFP, as a negative control, or sig-[HPV16E6LAMP]-IRES-GFP and sorted on the basis of GFP expression. Linking the sorting signal of lysosomal associated membrane protein-1 to HPV16E6 results in enhanced expression of HPV16E6 peptides in the context of MHC class II. Prior to the functional experiment, TCR transgenic T cells were kept in culture for a period of 6-8 weeks, and stimulated weekly with feeder cells and recombinant IL-2. Upon stimulation with T cell blasts transduced with sig-[HPV16E6LAMP]-IRES-GFP, resting TCR transgenic CD4+ T-cells were capable of producing IFN-γ, TNF-α, IL-5, IL-4 and IL-2 (Table 2). As expected, only low amounts of cytokines were produced upon stimulation with T cell blasts transduced with GFP control vector. No IL-10 production was observed after stimulation with T cell blasts transduced with either sig-[HPV16E6LAMP] or GFP (Table 2). These results show that in a TCR transgenic CD4+ bulk population both Th1 and Th2 cytokines are produced upon specific stimulation.

**Table 1:** Functional activity of 24.101 TCR transgenic CD4+ T cells as determined in a cytokine bead array assay.

	Th1 cytokines			Th2 cytokines		
	**IFNγ**	**TNFα**	**IL-2**	**IL-5**	**IL-4**	**IL-10**

**Unstimulated**	84	5.6	6.3	57	5.6	8.4
**GFP-control**	121	6.8	11	81	8.4	5.9
**Sig-[HPV16E6LAMP]-IRES-GFP**	>5000	162	2118	199	35	7.1

Target cells used in this assay were HLA-DP1+ T cell blasts or HLA-DP1+ T cell blasts carrying a gene encoding GFP or sig-[HPV16E6LAMP]-IRES-GFP. Amounts of cytokines produced are depicted as pg/ml. Results are representative for three different donors tested.

In conclusion, both Th1 and Th2 cytokines can be produced against HPV16E6 by transfer of MHC class II restricted HPV16E6 specific TCR ORFs in recipient CD4+ T cells.

### MHC class I restricted, HPV16E7 specific, TCR transgenic CD4+ T cells: TCR expression and functionality

Effective immunotherapy is dependent on the presence of both tumor specific CD4+ and CD8+ T cells. For this purpose it would be very attractive to use one and the same TCR which is functionally active in both CD4+ and CD8+ T cells. We investigated whether we could generate HPV specific CD4+ T cells using the MHC class I restricted HPV16E7_11-20 _specific TCR derived from CTL clone A9, which has previously been shown to be functionally active in recipient CD8+ T cells[19,26]. T cells carrying the HPV16E7 specific A9 TCR were previously shown to recognize the MHC class-I/peptide complex with intermediate avidity, reaching half-maximal lytic activity in the low nM range of peptide[25].

**Figure 4:**
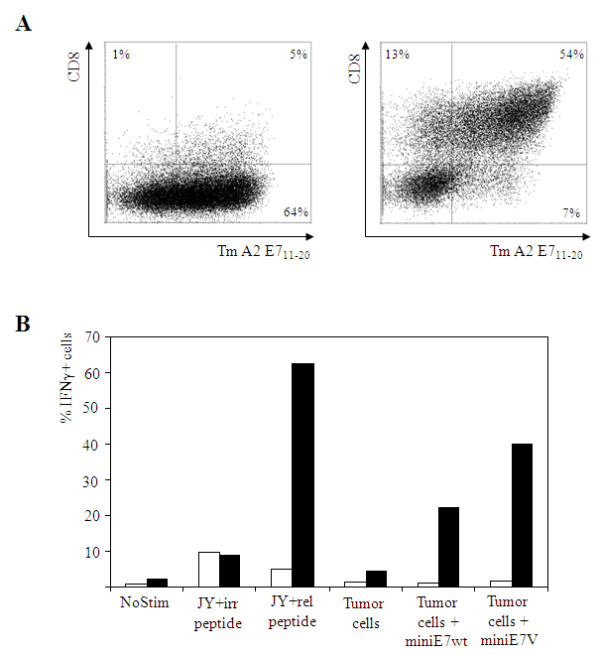
**Phenotypic analysis and functional activity of HPV16E7_(11-20) _specific A9 TCR transgenic CD4+ T cells**. (A) CD4+ T cells were transduced with cmTCRα-2A-cmTCRβ (left hand plot) and a proportion was co-transduced with CD8α-2A-CD8β (right hand plot). Cells were analyzed for binding to E7 tetramers and CD8 expression. (B) Functional activity of TCR-A9 transgenic CD4+ T cells as determined in an intracellular IFNγ staining. Effector cells did (black bars) or did not (open bars) carry the CD8αβ construct. Target cells used were HLA-A2 matched, non-autologous JY loaded with 1 μM irrelevant Influenza MP_(58-66) _or relevant HPV16E7_(11-20) _peptide; or the HLA-A2 matched non-autologous model tumor cell line melAKR either or not carrying a minigene construct encoding either HPV16E7_11-20wt _or HPV16E7_11-20V_. Results shown in panels A and B are representative for three different donors tested.

A bulk population of CD4+ T cells was transduced with cmTCRα-2A-cmTCRβ of the A9-TCR [26]. A proportion of these A9-TCR transgenic CD4+ T cells were transduced with retrovirus encoding for CD8α-2A-CD8β. TCR transgenic CD4+ T cells without the incorporation of CD8 were sorted based on tetramer binding, resulting in a population which was ~64% positive for tetramer binding. TCR transgenic CD4+ T cells transduced with CD8 were sorted based on tetramer binding and CD8 expression, resulting in a population which was ~54% positive for both tetramers and CD8 (Figure 4A).

In order to test functionality of the A9 TCR transgenic CD4+ T cells with or without CD8, an intracellular IFN-γ staining was performed. As shown in Figure 4B, approximately 6% of the CD4+ TCR transgenic T cells were capable of producing IFN-γ against JY cells exogenously loaded with relevant peptide. Some background IFN-γ production was observed after stimulation with JY cells loaded with irrelevant peptide. Addition of CD8 into CD4+ T cells resulted in IFN-γ production in 41% of the cells after stimulation with JY cells loaded with relevant peptide. As expected, some background IFN-γ production was observed after stimulation with JY cells loaded with irrelevant peptide. MelAKR cells encoding the minigene construct containing the HPV16E7wt epitope or HPV16E7V altered peptide ligand, with improved binding capacity to HLA-A2 were tested in an IFN-γ release assay. Only low percentages of IFN-γ production against the tumor cell lines was observed in TCR transgenic CD4+ T cells without the addition of CD8. In contrast, 22% of the cells transduced with both the TCR and CD8 showed IFN-γ production against the tumor cell line encoding HPV16E7wt and 40% against the tumor cell line encoding the improved HLA-A2 binding epitope HPV16E7V. As expected, unmodified tumor cells failed to stimulate IFN-γ production.

Subsequently, a CBA was performed to investigate which T helper cell populations were involved. TCR transgenic CD4+ T cells without the addition of CD8 showed hardly any cytokine production after stimulation with the tumor cell lines encoding the HPV16E7_11-20_wt and HPV16E7_11-20_V minigene (data not shown). In contrast, IFN-γ, TNF-α, IL-5, IL-4, IL-2 and low amounts of IL-10 were produced upon stimulation with the tumor cell lines encoding the HPV16E7wt and HPV16E7V constructs (Table 3). As expected, only low amounts of cytokines were produced upon stimulation with unmodified tumor cells. These results show involvement of both Th1 and Th2 responses. These experiments presented in Figure 4B and Table 3 do not proof that the A9 TCR is dependent on the (co-)expression of CD8. Formal proof could be obtained with tetramers that do not allow CD8 binding or blocking antibodies against CD8.

**Table 3:** Functional activity of A9 TCR transgenic CD4+ T cells as determined in a cytokine bead array assay.

	Th1 cytokines			Th2 cytokines		
	IFNγ	TNFα	IL-2	IL-10	IL-5	IL-4
**HPV16E7-negative**	180	10	5	4	52	7
**HPV16E7(11-20wt)**	1644	119	74	14	604	32
**HPV16E7(11-20V)**	>5000	591	893	52	2989	89

Target cells used in this assay were the HLA-A2 matched, non-autologous model tumor cell line melAKR, either or not carrying a minigene encoding for either HPV16E7_(11-20wt) _or HPV16E7_(11-20V)_. TCR transgenic T cells not co-expressing CD8αβ did not produce cytokine above threshold levels (data not shown). The amounts of cytokines produced, as determined in a cytokine bead array, are depicted as pg/ml. Results shown are representative for two different donors tested.

In conclusion, transfer of the MHC class I restricted TCR, derived from CTL clone A9, together with CD8 into recipient CD4+ T cells resulted in functional activity against peptide loaded target cells as well as (model-) tumor cells.

## Discussion

Adoptive transfer of TCR transgenic T cells is a promising strategy to treat patients suffering from malignancies. From clinical trials in melanoma patients it has become clear that both CD4+ and CD8+ T cells are required to induce effective antitumor responses[9,10]. It is assumed this holds true for other cancers as well. From our studies it is clear that a number of issues need to be addressed further. One is the specificity and affinity of the tumor specific T cells. Proper and functional expression of transgenic TCRs in recipient T cells leading to Th1 cytokine production and long-lasting anti-tumor activity is also desired.

Naturally occurring tumor antigen associated specific T cells often have low to intermediate affinity for their ligand, the peptide/MHC complex. In contrast to what one would expect because of the non-self nature of HPV antigens, the functional avidity of the original HPV16E6 specific T cell clone 24.101[34] and the 24.101-TCR transgenic T cells was low. The HPV16E7 specific T cell clone A9 was shown previously to recognize the MHC/peptide complex with intermediate avidity[25], as did wild-type and codon modified A9-TCR transgenic T cells[19,26]. Peptide based T cell stimulations may have favored the outgrowth of low to intermediate avidity T cells in vitro[35]. On the other hand high avidity T cells with high affinity TCRs may have been deleted from the TCR repertoire of these donors, either by thymic deletion or peripheral tolerance induction. To obtain high affinity TCRs for adoptive transfer purposes, tumor infiltrating lymphocytes isolated from patients suffering from HPV induced malignancies may be useful[36]. Other approaches may comprise of *in vivo *induction of tumor/virus specific T cells in humanized mouse models[37], *in vitro *affinity maturation using phage display technology[38,39], or the use of HLA mismatched donor material in which the antigen-presenting cells have been modified to express non-self MHC class I or class II antigen presenting molecules, thus addressing an unbiased T cell repertoire [40,41].

Transgenic TCR expression levels in human cells are often very low, necessitating antibiotic selection[42], enrichment or even cloning of TCR transgenic T cells before a sizeable population of TCR transgenic T cells can be tested for functional activity[19]. Enhancement of TCR expression levels can be accomplished using codon-modification alone[26,27] or in combination with cysteinization[20]. TCR gene transfer may also result in the undesired formation of mixed TCR dimers due to cross-pairing of the endogenous TCR chains with newly introduced TCR chains. Here we have used codon modification alone for TCR A9 and combined codon modification with cysteinization for TCR 24.101. In our studies on the HPV specific TCR 24.101 tetramers or multimers were unavailable for this particular MHC class II/peptide complex. Therefore we had to rely on GFP and NGFR marker expression, and on TCRVβ17 staining as a marker for expression of the transgenic TCR. It is unclear why only about one third of the GFP/NGFR transgenic bulk T cells in Figure 2 (gate R4; GFP/NGFR double positive cells) are positive for TCRVβ17 staining. After all in Figure 1 we showed that almost all (95%) of the GFP/NGFR transgenic Jurkat cells stained positive for TCRVβ17. The lack of staining with TCRVβ17 in GFP/NGFR transgenic bulk T cells may in part be explained by protein misfolding of the TCR or by promoter shut-down. The first explanation seems unlikely in view of the data in Jurkat T cells (Figure 1). The second explanation also seems unlikely since the marker NGFR was still expressed very well (Figure 2) and was under transcriptional control of the same MMLV-LTR promoter region. A more probable explanation may be that the 24.101 derived TCRα/β combination is a poor competitor for components of the CD3 complex[43,44]. As yet still unavailable tetramers and specific TCRVα antibodies would facilitate future research to address this issue.

The data presented in Figure 2, clearly indicated that the TCRβ derived from T cell clone 24.101 was able to cross-pair with endogenously present TCRα chains. The percentage naturally occurring TCRVβ17 positive T cells in that particular donor was 6.4% (GFP/NGFR double negative gate) and was expressed at high levels. In the GFP-/NGFR+ gate, approximately 18% of the cells were TCRβ17 transgenic (24% minus 6.4% endogenously expressed). Since no transgenic TCRα chain is present in the cells in this gate (R5) it shows that the TCRVβ17 behaved rather promiscuous. Approximately 31% (37% minus 6.4%) of the cells in the GFP+/NGFR+ gate are TCRβ17 transgenic. Low level expression and/or poor staining with the TCRβ17 specific antibodies may explain this finding, thus potentially giving an underestimation of the percentage TCR transgenic T cells. This could perhaps also explain why approximately 60% of cell incubated with relevant peptide loaded monocyte derived dendritic cells responded by the production of interferon, where only 36% of the cells stained positive with TCRVβ17 antibodies.

We have shown antigen recognition by 24.101 TCR transgenic T cells by using peptide loaded, or protein pulsed monocytes or alternatively CD4+ T cells expressing sig-HPV16E6-LAMP. In the case of TCR A9 transgenic T cells we used peptide loaded targets and a model-tumor cell line expressing either of two minigene constructs. Except for the peptide loaded target cells these approaches clearly show endogenous processing of the respective epitopes, albeit by target cells with relatively high expression of the presenting MHC molecules. The unavailability of cervical cancer target cells expressing the appropriate MHC restriction element at appreciable levels severely hampers functional analyses of HPV specific TCR transgenic T cells. HPV positive cancer cells often appear to have down-regulated MHC class I expression *ex vivo*, due to mutations in the beta 2-microglobulin, TAP or other genes involved in antigen presentation[45-47]. Future approaches in the field of HPV should therefore be focused on the generation of high avidity T cells able to recognize minute amounts of MHC class I on the cell surface of tumor cells and on high avidity MHC class II restricted T cells. Along these lines the HPV16E6 and HPV16E7 specific TCRs presented here should be tested in comparison to pre-existing or newly isolated HPV specific TCRs, like what has been done by others in the field of melanoma[27].

TCR transfer in unselected primary human CD4+ T cells might result in the development of suppressive T cell reactivity due to simultaneous transfer into regulatory T cells. IL-2 promotes the expansion of these regulatory T cells after several weeks of stimulation in vitro. However no IL-10 production was observed in our experiments after culturing the TCR transgenic T cells for 6-8 weeks in the presence of IL-2. If needed emerging regulatory T cells could be depleted from the population using CD25 antibody based MACS sorting. Alternatively, CD4+ T cell clones with a predetermined specificity and a favorable cytokine production profile might be used. Previous reports have shown that both Th1 and Th2 cells mediate anticancer functions[48] but IFNγ secreting Th1 cells appeared to be more effective in this role[48,49]. Therefore, immunotherapy using TCR transgenic CD4+ Th1 cells may be more desirable in a clinical setting. Cytokine production profiles of 24.101 and A9 TCR transgenic CD4^+ ^T-cells indicate that both Th1 and Th2 subsets were represented. To obtain a Th1 population, CD4+ T cells could be sorted or enriched using an interferon-γ catch assay. Alternatively transgenic CD4+ T cells could be polarized towards a Th1 cytokine production profile by culturing them in the presence of IL-12 and anti-IL-4, as has been shown in mice [49].

Conflicting data have been reported on the occurrence of autoimmunity *in vivo*. In a previously published study no signs of autoimmune pathology were observed in mice receiving TCR transgenic T cells in combination with antigen specific vaccination[50]. However, more recent data show lethal autoimmune pathology under conditions that promote expansion of TCR transgenic T cells more strongly[51,52]. Thus far no signs of severe autoimmunity have been observed in the first clinical trial conducted[53]. However, on the basis of data obtained in mouse models the use of strategies to limit the formation of mixed TCR dimers are important to explore. Molecular engineering approaches have been published to reduce the formation of mixed TCR dimers, including the use of single chain TCRs[54,55], cysteinization of the constant TCRα and TCRβ chains[28], murinization of TCR constant domains[56], and inter-chain conversion of the constant domains, referred to as the hole-into-knob approach[57]. Cellular approaches include the use of γδ-T cells[58], NK cells[59], or T cell clones with predetermined specificities[60]. The use of small interfering RNAs, specifically inhibiting the expression of the endogenous TCRα and/or TCRβ chains, may be another strategy to prevent cross-pairing[61]. Careful analysis of different engineering approaches should be performed to determine which is best applicable. In the field of HPV there is a continuing need for high affinity TCRs to functionally compare these in appropriate *in vitro *and *in vivo *model systems.

## Conclusion

In this paper we show that it is feasible to express functional TCRs that recognize HPV antigens presented by MHC class I and class II antigens in CD4+ T cells. A combination of high avidity TCR transgenic HPV16 specific CD4+ and CD8+ T cells might be ideal for the treatment of patients suffering from cervical cancer and other HPV induced malignancies.

## Abbreviations

CxCa: cervical cancer; CTL: cytotoxic T lymphocyte; GFP: green fluorescence protein; HPV: Human Papilloma Virus; IFN: Interferon; IRES: internal ribosomal entry site; NGFR: nerve growth factor receptor; TCR: T cell receptor.

## Competing interests

The authors declare that they have no competing interests.

## Authors' contributions

KBJS performed experiments, analyzed and interpreted the data and wrote the manuscript, AWT and JJR performed experiments, MH provided experimental devices, MHMH and SHB provided experimental devices and revised the paper, CJLMM revised the paper, EH designed the project, analyzed and interpreted the data, and wrote and revised the manuscript.

All authors read and approved the final manuscript.
